# High-Throughput Screening of Co-Protoporphyrin IX-Binding Proteins for Enhanced Hydrogen Production

**DOI:** 10.3390/molecules31020346

**Published:** 2026-01-19

**Authors:** Nicholas Ryan Halloran, Mohammad Imtiazur Rahman, Roman Christopher Fabry, Abesh Banerjee, Giovanna Ghirlanda

**Affiliations:** School of Molecular Sciences, Arizona State University, Tempe, AZ 85287, USA

**Keywords:** artificial metalloenzymes, hydrogen evolution, cobalt porphyrin, catalysis, high-throughput screening

## Abstract

Artificial metalloenzymes incorporating cobalt protoporphyrin IX (Co-PPIX) are promising for sustainable hydrogen production; however, slow protein preparation and a lack of suitable detection methods limit the systematic optimization of their catalytic performance. Here, we report a streamlined workflow that combines the direct in vivo incorporation of Co-PPIX into cytochrome *b*_562_ (cyt *b*_562_) variants with a colorimetric assay for hydrogen evolution, scalable to hundreds of mutants. We screened 103 members of a mutant library and selected the variant Co-Mut25, which displayed activity double than wild type on the screen, and produced over 70% more hydrogen than WT as assessed by gas chromatography. This approach enables the rapid and scalable identification of high-performing cobalt–protein catalysts and expands the toolkit for artificial hydrogenase development.

## 1. Introduction

Artificial metalloenzymes can be created by incorporating organometallic catalysts into protein frameworks. They serve as bridges between traditional molecular catalysts and enzymes and offer the advantage of broadening the range of chemical reactions beyond those found in natural enzymes. For instance, natural heme-binding proteins have been engineered to perform reactions that are absent in nature, such as carbene and nitrene transfer and Kemp elimination, achieved through rational design and directed evolution [1,2,3,4,5,6,7,8,9] or by substituting the central iron with other metals to explore different chemistries [10,11,12,13,14,15].

Recently, our group and others reported artificial metalloenzymes containing cobalt protoporphyrin IX, demonstrating their activity in hydrogen production and, more recently, in carbon dioxide reduction under photocatalytic and electrocatalytic conditions [3,16,17,18,19,20,21,22,23,24]. These constructs leverage the inherent reactivity of organometallic cobalt porphyrins [25,26,27,28,29,30] and address challenges, such as low solubility, synthetic complexity, and difficulties in modifying primary and secondary coordination spheres to optimize their reactivity. The protein scaffold, whether derived from natural proteins or constructed from scratch, modulates the reactivity of the organometallic center, provides adjustable primary and secondary coordination spheres, and protects the organometallic center from degradation [31,32,33,34,35,36,37,38].

The activity of WT scaffolds can be influenced by mutations in both the primary and secondary spheres, as demonstrated by rationally designed mutants [3,16,22,23,24]. For instance, substituting the coordinating His102 and Met7 in Co-cyt *b*_562_ with Ala, Asp, or Glu increased TON substantially. Modeling indicates that Asp can relay protons to cobalt, whereas Ala likely facilitates water coordination [16,22]. However, the limited number of mutants explored per scaffold was unlikely to yield significant improvements or generalizable insights.

The application of a directed evolutionary strategy to identify high-performing mutants from extensive libraries is impeded by the labor-intensive process of preparing recombinant proteins. This process involves overexpression of the apo protein, which frequently results in a hemin-bound form, followed by purification and reconstitution with cobalt protoporphyrin IX (Co-PPIX). Another significant challenge is the lack of a sensitive and scalable high-throughput method for evaluating the hydrogen production efficiency of metalloproteins. Molecular hydrogen production was accurately quantified using gas chromatography, which is not easily scalable. Current methodologies, such as coupled enzymatic assays with hydrogenase, are often labor-intensive, exhibit limited sensitivity, and necessitate conditions that are incompatible with our expression system [31,32,33,34].

**Scheme 1:**
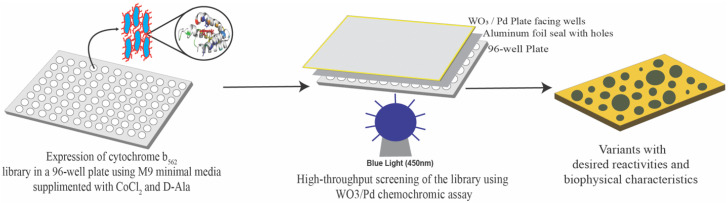
Summary of work.

In this study, we present a comprehensive workflow for the preparation and screening of a Co-cyt *b*_562_ mutant library. This process is based on the direct in vivo incorporation of Co-PPIX, facilitated by co-expression of the heme transporter ChuA or metabolic biosynthesis in engineered *E. coli*. The activities of the mutants were evaluated using a colorimetric method. Employing this approach, we effectively screened a library generated by error-prone PCR, which led to the identification of Co-Mut25, a mutant that demonstrated enhanced hydrogen production activity compared to the wild type (Scheme 1).

## 2. Results 

### 2.1. Optimization of Protein Expression

Metalloporphyrin-substituted heme proteins can be produced in the *E. coli* BL21 strain either through intracellular reconstitution with the desired porphyrin, or via Co-PPIX biosynthesis in the case of cobalt. In the first approach, a plasmid encoding the target protein was co-transformed with a plasmid encoding the outer-membrane heme transporter ChuA [35,36]. To inhibit heme biosynthesis and incorporation, cells were cultivated in minimal medium without iron, and the growth medium was supplemented with the desired porphyrin simultaneously with IPTG induction, leading to direct incorporation of the metalloporphyrin-substituted protein. ChuA’s promiscuity extends to metalloporphyrins such as Co^3+^, Zn^2+^, Ir^3+^, and Mn^3+^ PPIX, and it accommodates alterations in the porphyrin ring, generally achieving over 90% incorporation efficiency with high yields [2,13,37,38].

We adapted this method for high-throughput protein expression to facilitate the extraction of pure proteins incorporating the desired metalloporphyrin from the periplasmic space, thereby eliminating the need for purification and enhancing the incorporation efficiency. Briefly, protein localization is directed to the periplasm using a Sec translocation tag, which is cleaved simultaneously with translocation [35]. SecB, a component of the Sec translocase system, binds to the Sec signal sequence and guides protein translocation in its unfolded state [39,40]. Interestingly, we discovered that the periplasmic fraction contained Co-cyt *b*_562_, whereas the cytoplasm contained small amounts of Fe(II)-PPIX bound to cyt *b*_562_, likely because of incomplete translocation into the periplasm and the reducing environment of the cytoplasm. The species were identified by UV–Vis spectroscopy based on the characteristic Soret peaks at 416 nm for Fe(III) cyt *b*_562_, 425 nm for Fe(II) cyt *b*_562_, and 427 nm for Co(III) cyt *b*_562_, along with distinctive Q bands (Figure S1) [22]. The second protocol for overexpressing Co-cyt *b*_562_ exploited the ability of *E. coli* BL21(DE3) to biosynthesize cobalt protoporphyrin IX (Co-PPIX) when cultured in a medium supplemented with high concentrations of cobalt and the heme biosynthesis precursor δ-aminolevulinic acid (δ-ALA) [41]. In BL21(DE3), which lacks the RcnA efflux system, cobalt accumulates intracellularly and is inserted into free-base porphyrins by promiscuous *E. coli* ferrochelatase (EcHemH). Cofactor incorporation occurs concurrently with hemoprotein expression, allowing the protein to sequester cobalt as Co-PPIX and mitigate the toxicity of free cobalt.

We used two distinct methodologies to express a library of mutants generated by error-prone PCR. In both approaches, the holo protein was localized within the periplasmic space. Individual colonies were cultivated in deep-well 96-well plates, and the periplasmic fraction was released through cold osmotic shock. The initial characterization of the mutants was conducted using UV–Visible spectroscopy on a plate reader to determine the concentration of Co-cyt *b*_562_ by monitoring the Soret band at 427 nm, while the protein concentration was independently assessed at 280 nm. The ratio of absorbance at 427 nm to that at 280 nm provided an estimate of the relative Co-PPIX incorporation efficiency for each mutant. The plates were subsequently used for further characterization and analysis.

### 2.2. High-Throughput Assay Development

**Figure 1:**
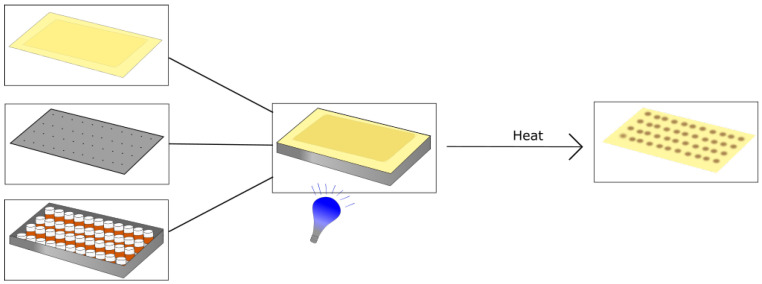
Diagram of WO_3_/Pd reaction setup in 96 well plate.

Our colorimetric screen relies on tungsten (VI) oxide (WO_3_), a metal oxide that undergoes a color change from yellow to blue upon partial reduction from W^6+^ to W^5+^ in the presence of hydrogen. This gasochromic effect, typically catalyzed by surface-bound palladium or platinum, which facilitates H_2_ dissociation, has been extensively studied for industrial hydrogen leak detection. However, many WO_3_-based assays require inert conditions, elevated temperatures, or specialized instrumentation, which limit their applicability in biological or photocatalytic assays. We adapted this assay for reactions occurring in aerobic and aqueous environments under continuous illumination, which interferes with detection. We deposited WO_3_ nanoparticles on a glass plate support and added a solution of Pd(II) to the dry coating to prepare Pd/WO_3_ plates. The plates were placed over a 96-well plate covered with foil sealing film and pierced in correspondence with each well (Figure 1).

**Figure 2:**
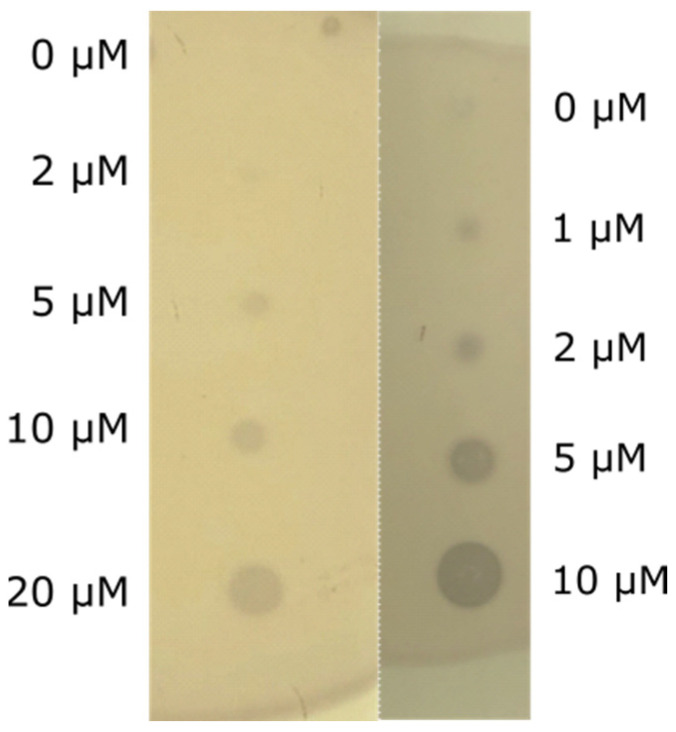
WO_3_/Pd plates after exposure to increasing concentrations of Co-PPIX. Control reactions with increasing Co-PPIX concentrations in the wells showed that the color change increases with increased H_2_ production. (**Left**): Free Co-PPIX, (**Right**): Co-PPIX-M1; Numbers refer to concentration of Co-PPIX in the reaction wells.

The Pd/WO_3_ plates were evaluated for their response to varying levels of hydrogen gas. Control reactions using increasing concentrations of Co-PPIX showed a corresponding increase in H_2_ production, as evidenced qualitatively by the size and intensity of the blue coloration above each well after heating (Figure 2). We compared the activity of free Co-PPIX with that of Co-M1, a peptide that catalyzes hydrogen production with an efficiency approximately eight times higher than that of free Co-PPIX under similar conditions [42]. Consistent with previous observations, the M1-bound form exhibited higher activity than the free porphyrin, as indicated by the darker and larger colorimetric responses. A lower detection limit was established for 1 µM protein-bound Co-PPIX, which produced a visible color change. The detection limit of gas chromatography was determined to be approximately 20–25 nmol of H_2_, equivalent to ~0.3% H_2_ in the 180 µL headspace of a 96-well plate (Figure 2).

### 2.3. Isolation and Validation of Mut25

**Figure 3:**
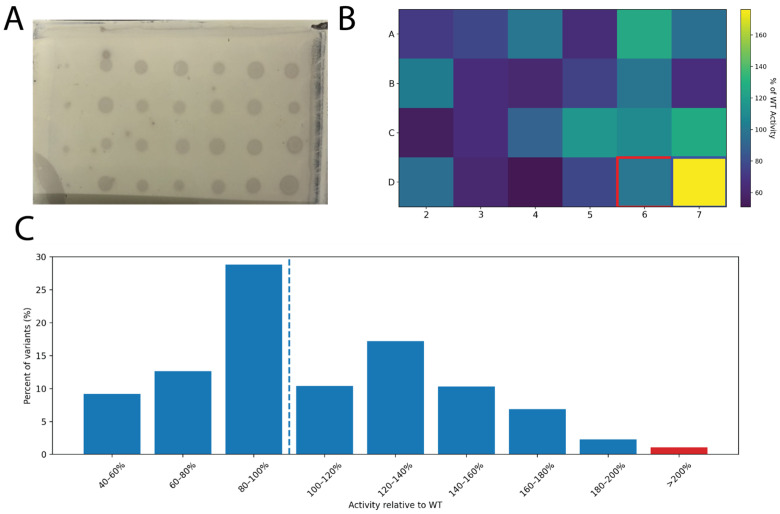
High-throughput screening of cytochrome *b*_562_ variants using a WO_3_/Pd colorimetric hydrogen evolution assay. (**A**) Raw image of a representative screening plate showing WO_3_/Pd darkening induced by hydrogen evolution. (**B**) Corresponding heat map of WT-normalized activity (WT = 100%), with WT (D6) and Mut25 (D7) positions highlighted. (**C**) Histogram of WT-normalized activities pooled across all experiments, showing a distribution centered near WT (dotted line) with a pronounced high-activity tail and Mut25 as the sole > 200% WT outlier.

The hydrogen evolution activity of the mutants was evaluated by directly screening the crude periplasmic fractions using WO_3_/Pd plates. Briefly, aliquots (200 µL) containing 5 µM Co-PPIX-bound protein, 1 mM [Ru(bpy)_3_]^2+^, and 200 mM ascorbic acid were dispensed into a fresh 96-well plate. The plates were sealed, overlaid with a WO_3_/Pd sensor glass, irradiated with a 55 mW cm^−2^ blue LED array for 10 min, and left in the dark for 15 min. After oven-drying the plate, the wells corresponding to the largest circles and the deepest blue hue exhibited the highest H_2_ evolution. The plate images were analyzed to estimate the amount of hydrogen produced from the raw WO_3_/Pd colorimetric intensities, which were normalized to WT (set to 100%) within each plate. Figure 3A,B show the raw image and heatmap of activity values at their corresponding plate positions for a typical plate, revealing substantial variations among variants and highlighting WT (D6) and Mut25 (D7). To assess the overall performance landscape of the library, WT-normalized activities from all screening experiments were pooled and summarized as a histogram (Figure 3C). Normalization within each experiment to the mean WT activity corrects for inter-experiment variability and allows direct comparison across plates. The resulting distribution is centered near WT activity, with approximately 40% of variants falling within 80–120% of WT. Approximately 20% display enhancements greater than 140% of WT. Mut25 consistently occupies the extreme upper end of the distribution and is the only variant to reproducibly exceed 194 ± 0.17% of WT activity (~1%), and qualitatively outperformed WT- at every purification stage (whole cells, periplasm, and purified protein (Figure S2)).

Sequence analysis identified seven point-mutations within the gene encoding Mut25, four of which were silent. Two mutations correspond to two amino acid substitutions, A20V and D21N, using WT numbering (PDB 2BC5). An A to T mutation resulted in the conversion of the native lysine codon (Lys 15) to the Amber stop codon “TAG.” Note that a serine had been added at the N terminus to improve the yield of TEV cleavage during library construction. Although Mut25 should be truncated at position 15, the full-length protein was successfully obtained following expression and purification using the same protocol as for WT, albeit in small quantities (yield < 1 mg for 1 L fermentation). Analytical HPLC comparison indicated that Mut25 eluted at a solvent composition 1% higher in B than the wild type (WT) (Figure S4). MALDI analysis of HPLC-purified Mut25 revealed a mass of 11,969.14 Da, consistent with the presence of A20V, D21N, and the additional serine residue at the N terminus (Figure S5).

**Figure 4:**
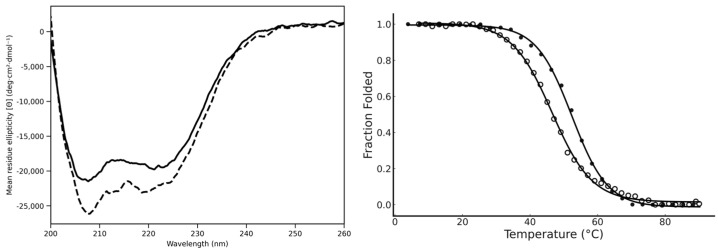
CD spectra (**left**) of Mut25 in the apo (solid line) and bound (dashed line) states and thermal denaturation curves (**right**) of Mut25 in the apo (open circles) and bound (filled circles) states. Conditions: Mut25 8 µM in 50 mM sodium phosphate, pH 7.5; CoPPIX 20 µM.

We characterized the secondary structure and stability of Mut25 in the apo and CoPPIX-bound states using circular dichroism. Both states were α-helical, as shown by the minima at 208 and 222 nm (Figure 4), consistent with AlphaFold 3 modeling (Figure S6). Binding to CoPPIX resulted in minimal changes in this region, indicating that the incorporation of the cofactor had little effect on the structure of the protein at room temperature. Thermal denaturation curves obtained by monitoring the loss of intensity of the 222 nm peak at increasing temperatures show that the holo state is more stable than the apo state: the apparent midpoint of the thermal denaturation, *T*_m_, increases from 46.5 °C to 52.3 °C upon binding of the porphyrin, consistent with prior observations on WT and its mutants [22].

**Figure 5:**
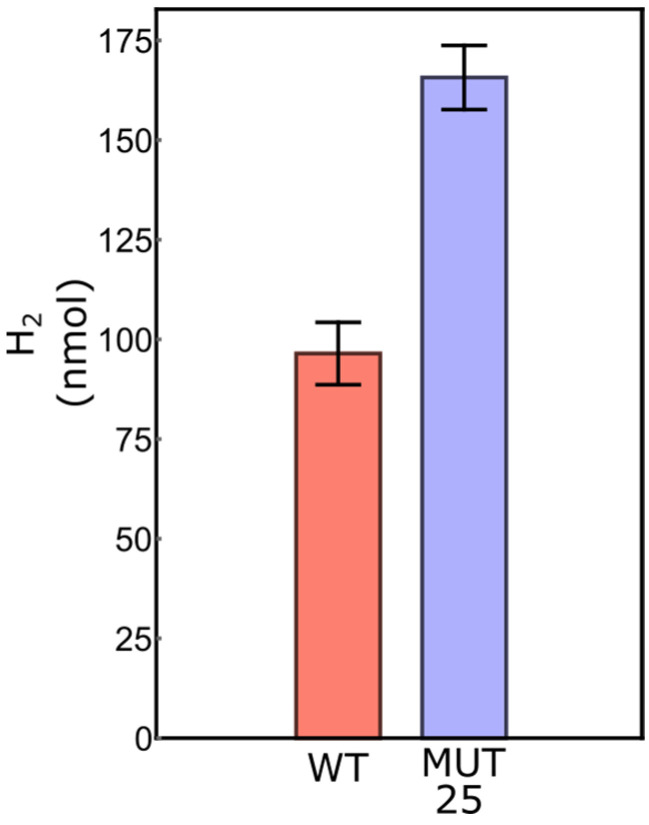
Hydrogen evolution by WT cyt *b*_562_ (red) and Mut25 (blue). Conditions: 0.86 µM Co-PPIX, 860 µM [Ru(bpy)_3_]^2+^, 43 mM ascorbic acid, and 3 µM protein in sodium phosphate buffer pH 6.5 at 25 °C after 30 min of illumination; samples were run in triplicate.

Next, we verified the results of the high-throughput activity screening by comparing the activity of Co-Mut25 with that of the wild type (WT) using gas chromatography. The proteins were expressed and purified in their apo form, reconstituted with Co-PPIX in vitro, and assessed in triplicates at a concentration of approximately 1 mM. Gas chromatographic analysis after 30 min (0.86 µM CoPPIX, 5 µM protein, pH 6.5, 25 °C, 55 mW cm^−2^ blue light, 30 min) confirmed a 70% increase, with Mut25 producing 165 ± 4 nmol H_2_ compared to 96 ± 3 nmol for the wild type (Figure 5). Because hydrogen evolution did not reach a steady state or plateau within the 30 min, we report the H_2_ yield at 30 min, which serves as a measure of the extent of catalysis under these standardized conditions.

## 3. Discussion

This study provides a workflow for the direct expression of cobalt-substituted heme-binding protein libraries and screening of photocatalytic hydrogen production. To date, the direct incorporation of cobalt in heme-binding proteins has been demonstrated for individual proteins but has not been adapted for high-throughput use [43,44]. Similarly, the screening of catalysts for hydrogen production has been limited to hydrogenase mutants, which produce hydrogen under different conditions and in larger amounts [32]. High-throughput screening under photocatalytic conditions has been demonstrated for organometallic catalysts under conditions incompatible with protein expression [33]. Furthermore, our method requires minimal equipment, provides high sensitivity, and is compatible with various conditions. Comparative analysis between the WO_3_/Pd colorimetric assay and gas chromatography confirmed the assay’s reliability and high sensitivity as a surrogate measure of hydrogen evolution.

**Figure 6:**
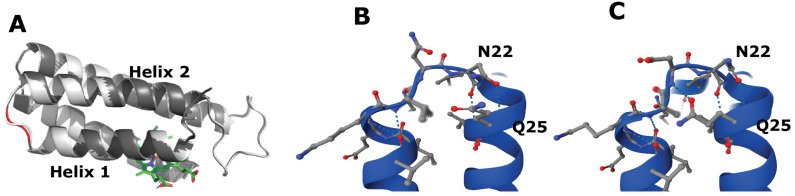
AlphaFold3 generated models of WT cyt *b*_562_ and Mut 25. (**A**) Overlay of WT (light grey) and Mut25 (dark grey, mutations in red). The mutations are located in the first loop and affect the hydrogen bonding network: In Mut25 (Panel (**B**)) the A20V mutation perturbs side chain to backbone hydrogen bonding observed for N22 and Q25 in WT (Panel (**C**)).

Using this workflow, we discovered a mutant of cyt *b*_562_ that consistently displayed the highest activity on plate assay. Further characterization by gas chromatography confirmed that the purified Mut25 catalyzes photoinduced hydrogen production with H_2_ yield at 30 min 70% higher than WT. Mutations in Mut25 (A20V, D21N using WT nomenclature) were located outside the immediate axial coordination sphere of the cobalt center, suggesting that subtle changes in the secondary shell environment can modulate catalytic efficiency. Comparison of models of Mut25 and of WT cyt *b*_562_ generated by AlphaFold3 reveal high similarity (Figure 6), but indicated that these mutations affect the sidechain to backbone hydrogen bonding observed at the N terminus of helix 2 in WT (N22, Q25); in addition the mutations may reposition helix 1, which bears the axial Met7 ligand, by interacting with neighboring Val16. These findings align with previous reports that modifications beyond the primary coordination sphere can significantly influence peroxidase activity in mutants of Fe(III)PPIX bound cyt *b*_562_ through a surprisingly large effect on the redox potential of the Fe(II)/Fe(III) couple [41,45].

Mut25 also comprises the amber codon “TAG,” a termination codon, at position 19; however, we recovered the full-length protein, as assessed by MALDI spectrometry (Figure S5). There is evidence that the TAG codon can be translated as near-cognate lysine or glutamine in non-modified strains at a non-negligible frequency [46,47]. Furthermore, the presence of aminoglycoside antibiotics during protein expression may increase the misreading error rate [48]. A read-through of the amber codon also explains the significantly reduced yield of pure protein compared with that of the wild type.

## 4. Materials and Methods

Plasmid Construction and Protein Expression: Detailed protocols are described in SI. Briefly, cyt *b*_562_ was cloned into plasmid pET26b (Addgene, Watertown, MA, USA) containing a second translocation signal, His-tag, and TEV cleavage site [22]. Two methods were used for Co-PPIX incorporation. *ChuA* Transporter Method: the ChuA gene from the original kanamycin-resistant plasmid (pChuA #42539, Addgene, USA) was transferred into T7p14_15His_deGFP (ampicillin-resistant) to ensure double selection with antibiotics of the co-transformed cells. Plasmids encoding cyt *b*_562_ and ChuA were co-transformed into *E. coli* BL21 DE3 (New England Bioloabs, Ipswich, MA, USA) under double antibiotic selection. Cells were grown in M9 medium and induced with 1 mM IPTG at OD600. Cells were pelleted, washed with M9 medium, and a solution of Co-PPIX (Sigma-Aldrich, St. Louis, MO, USA) to a final concentration of 5 mM was added to the suspension. After 30 min, the periplasmic fraction was isolated using cold osmotic shock with 1 mM MgCl_2_ and purified using affinity chromatography on Ni-NTA resin (Cytiva, Marlborough, MA, USA). Fractions containing the desired proteins were pooled, treated with TEV protease, and re-purified using affinity chromatography. Metabolic biosynthesis methods. The second method involved directly expressing the Co-PPIX-bound protein in *E. coli* BL21(DE3) by supplementation with cobalt chloride and δ-aminolevulinic acid in the growth medium M9. After induction at OD600, holo protein was purified from the periplasmic fraction, as described above.

Library Generation and Expression: A library of random mutants of Sec-His-cytochrome *b*_562_ was generated using the Agilent GeneMorph II EZClone Domain Mutagenesis Kit (Santa Clara, CA, USA) by error-prone PCR using primers designed to protect tagging sites and stop codons (Table S4). Error-prone PCR was performed following the manufacturer’s instructions, selecting the initial DNA template and PCR cycles to obtain 4.5–9.0 bp mutants per kb gene as mutation frequency, corresponding to 2–3 bp mutations per colony for a 318 bp gene (100 ng plasmid, 25 cycles of amplification). An annealing temperature of 60 °C was used for amplification during error-prone PCR. The amplicon from error-prone PCR was used as a megaprimer for RF cloning of the Sec-His-cytochrome *b*_562_ variants. The PCR product was purified using a Promega DNA Cleanup kit (Madison, WI, USA), transformed into a cloning strain, and the cells were plated on agar. All colonies were collected and plasmid DNA was extracted using the Qiagen Spin Miniprep Kit (Hilden, Germany).

High-throughput screening for hydrogen production activity was performed using WO3/Pd-coated glass plates, as described in the Supplementary Information. Molecular hydrogen generated under photocatalytic conditions induced a measurable darkening reaction on these plates. The plates were imaged and analyzed with the MicrobeJ plug-in in Fiji [49,50].

Hydrogen evolution: The CoPPIX stock solution was formulated by dissolving the solid compound in dimethyl sulfoxide (DMSO) at a concentration of 1 mg/100 µL, followed by 1:1000 dilution in 500 mM sodium phosphate buffer (pH 6.9). The concentration was verified using UV–Vis spectroscopy (Perkin Elmer, Shelton, CT, USA). CoPPIX was then introduced to a five-fold molar excess of M1 protein and incubated at ambient temperature for 30 min. Tris(bipyridine)ruthenium(II) chloride ([Ru(bpy)3]^2+^) was dissolved in 500 mM sodium phosphate buffer (pH 6.9) to function as a photosensitizer, whereas ascorbic acid was dissolved to act as an electron donor. The reaction mixtures were prepared in Eppendorf tubes (Hamburg, Germany) at final concentrations of 5 µM M1, 1 µM CoPPIX, 200 mM ascorbic acid, and 1 mM [Ru(bpy)3]^2+^ in 500 mM sodium phosphate buffer (pH 6.5). These mixtures were subsequently transferred to 2 mL glass vials, sealed, and purged with nitrogen gas. The samples were placed in a HepatoChem Temperature-Controlled PhotoRedOx Box (Cambridge, MA, USA), maintained at 25 °C through water circulation, and illuminated with an EvoluChem 55 mW/cm^2^ blue LED. The hydrogen evolved from the reactions was quantified using a gas chromatograph (SRI Instruments, Torrance, CA, USA) equipped with a thermal conductivity detector (TCD) and a 3′ × 1/8″ molecular sieve 5 Å column. Argon was employed as the carrier gas and the TCD temperature was maintained at 100 °C to facilitate the detection of hydrogen, oxygen, and nitrogen gases in the samples. At one-hour intervals, the reaction vials were removed from the light source, inverted, and allowed to stand on the bench for 15 min. A 250 µL Hamilton gas-tight syringe, purged with argon, was used to inject 100 µL of argon into each inverted vial. Immediately thereafter, 100 µL of headspace gas was withdrawn and injected into the gas chromatography column. This procedure was repeated for all the samples before they were returned to the photoreactor. All gas chromatography experiments were conducted in triplicate unless otherwise specified.

The total amount of H_2_ produced was calculated using a standard curve (Figure S7).

## 5. Conclusions

The development of a high-throughput screening platform for Co-PPIX-binding proteins addresses significant bottlenecks in the directed evolution of artificial metalloenzymes for hydrogen production. By combining in vivo Co-PPIX incorporation with a sensitive WO_3_/Pd-based colorimetric assay, we enabled the rapid and scalable evaluation of hydrogen evolution activity directly from crude periplasmic extracts, bypassing the laborious purification and gas chromatographic quantification traditionally required. The assay’s ability to detect as little as 25 nmol H_2_ with a straightforward visual readout under aerobic, aqueous conditions represents a considerable advancement in accessibility and throughput for screening metalloprotein libraries. The successful identification of the Mut25 variant, which exhibited a 70% enhancement in hydrogen production relative to wild-type cyt *b*_562_, validates the utility of this approach. We plan to expand this approach by characterizing and sequencing a larger number of mutants. Given the increasing interest in cobalt-substituted heme-binding proteins for various catalytic applications, we hope that our workflow will find broad use.

Future efforts will focus on expanding the mutant library size and integrating computational modeling to comprehensively map the structure–activity relationships. This approach promises to provide further insights into how distal residues influence cobalt porphyrin reactivity, ultimately guiding rational design and directed evolution strategies for enhanced artificial hydrogenases. Additionally, the simplicity and stability of the WO_3_/Pd plates position this assay as a broadly applicable tool for distributed research and educational settings.

## Data Availability

The original contributions presented in this study are included in the article/Supplementary Material. Further inquiries can be directed to the corresponding author(s).

## Supplementary Materials

The following supporting information can be downloaded at: https://www.mdpi.com/article/10.3390/molecules31020346/s1. References [16,22,43,44,51] are cited in the Supplementary Materials.

## References

[1] Bhattacharya S., Margheritis E.G., Takahashi K., Kulesha A., D’Souza A., Kim I., Yoon J.H., Tame J.R.H., Volkov A.N., Makhlynets O.V. (2022). NMR-Guided Directed Evolution. Nature.

[2] Bordeaux M., Singh R., Fasan R. (2014). Intramolecular C(Sp3)H Amination of Arylsulfonyl Azides with Engineered and Artificial Myoglobin-Based Catalysts. Bioorganic Med. Chem..

[3] Deng Y., Dwaraknath S., Ouyang W.O., Matsumoto C.J., Ouchida S., Lu Y. (2023). Engineering an Oxygen-Binding Protein for Photocatalytic CO_2_ Reductions in Water. Angew. Chem. Int. Ed..

[4] Lin Y.-W., Yeung N., Gao Y.-G., Miner K.D., Tian S., Robinson H., Lu Y. (2010). Roles of Glutamates and Metal Ions in a Rationally Designed Nitric Oxide Reductase Based on Myoglobin. Proc. Natl. Acad. Sci. USA.

[5] Liu Z., Arnold F.H. (2021). New-to-Nature Chemistry from Old Protein Machinery: Carbene and Nitrene Transferases. Curr. Opin. Biotechnol..

[6] Moore E.J., Fasan R. (2019). Effect of Proximal Ligand Substitutions on the Carbene and Nitrene Transferase Activity of Myoglobin. Tetrahedron.

[7] Sigman J.A., Kwok B.C., Lu Y. (2000). From Myoglobin to Heme-Copper Oxidase: Design and Engineering of a Cu _B_ Center into Sperm Whale Myoglobin. J. Am. Chem. Soc..

[8] Tinzl M., Diedrich J.V., Mittl P.R.E., Clémancey M., Reiher M., Proppe J., Latour J.-M., Hilvert D. (2024). Myoglobin-Catalyzed Azide Reduction Proceeds via an Anionic Metal Amide Intermediate. J. Am. Chem. Soc..

[9] Vargas D.A., Ren X., Sengupta A., Zhu L., Roy S., Garcia-Borràs M., Houk K.N., Fasan R. (2024). Biocatalytic Strategy for the Construction of Sp3-Rich Polycyclic Compounds from Directed Evolution and Computational Modelling. Nat. Chem..

[10] Cai Y.-B., Yao S.-Y., Hu M., Liu X., Zhang J.-L. (2016). Manganese Protoporphyrin IX Reconstituted Myoglobin Capable of Epoxidation of the CC Bond with Oxone^®^. Inorg. Chem. Front..

[11] Dydio P., Key H.M., Nazarenko A., Rha J.Y.-E., Seyedkazemi V., Clark D.S., Hartwig J.F. (2016). An Artificial Metalloenzyme with the Kinetics of Native Enzymes. Science.

[12] Gengenbach A., Syn S., Wang X., Lu Y. (1999). Redesign of Cytochrome c Peroxidase into a Manganese Peroxidase:  Role of Tryptophans in Peroxidase Activity. Biochemistry.

[13] Key H.M., Dydio P., Liu Z., Rha J.Y.-E., Nazarenko A., Seyedkazemi V., Clark D.S., Hartwig J.F. (2017). Beyond Iron: Iridium-Containing P450 Enzymes for Selective Cyclopropanations of Structurally Diverse Alkenes. ACS Cent. Sci..

[14] Low D.W., Abedin S., Yang G., Winkler J.R., Gray H.B. (1998). Manganese Microperoxidase-8. Inorg. Chem..

[15] Mann S.I., Nayak A., Gassner G.T., Therien M.J., DeGrado W.F. (2021). De Novo Design, Solution Characterization, and Crystallographic Structure of an Abiological Mn–Porphyrin-Binding Protein Capable of Stabilizing a Mn(V) Species. J. Am. Chem. Soc..

[16] Alcala-Torano R., Halloran N., Gwerder N., Sommer D.J., Ghirlanda G. (2021). Light-Driven CO_2_ Reduction by Co-Cytochrome *B*_562_. Front. Mol. Biosci..

[17] Alvarez-Hernandez J.L., Salamatian A.A., Han J.W., Bren K.L. (2022). Potential- and Buffer-Dependent Selectivity for the Conversion of CO_2_ to CO by a Cobalt Porphyrin-Peptide Electrocatalyst in Water. ACS Catal..

[18] Edwards E.H., Le J.M., Salamatian A.A., Peluso N.L., Leone L., Lombardi A., Bren K.L. (2022). A Cobalt Mimochrome for Photochemical Hydrogen Evolution from Neutral Water. J. Inorg. Biochem..

[19] Edwards E.H., Bren K.L. (2020). Light-Driven Catalysis with Engineered Enzymes and Biomimetic Systems. Biotechnol. Appl. Biochem..

[20] Firpo V., Le J.M., Pavone V., Lombardi A., Bren K.L. (2018). Hydrogen Evolution from Water Catalyzed by Cobalt-Mimochrome VI*a, a Synthetic Mini-Protein. Chem. Sci..

[21] Kleingardner J.G., Kandemir B., Bren K.L. (2014). Hydrogen Evolution from Neutral Water under Aerobic Conditions Catalyzed by Cobalt Microperoxidase-11. J. Am. Chem. Soc..

[22] Sommer D.J., Vaughn M.D., Clark B.C., Tomlin J., Roy A., Ghirlanda G. (2016). Reengineering Cyt *B*_562_ for Hydrogen Production: A Facile Route to Artificial Hydrogenases. Biochim. Biophys. Acta (BBA)-Bioenerg..

[23] Sommer D.J., Vaughn M.D., Ghirlanda G. (2014). Protein Secondary-Shell Interactions Enhance the Photoinduced Hydrogen Production of Cobalt Protoporphyrin IX. Chem. Commun..

[24] Labidi R.J., Faivre B., Carpentier P., Perard J., Gotico P., Li Y., Atta M., Fontecave M. (2024). Light-Activated Artificial CO_2_-Reductase: Structure and Activity. J. Am. Chem. Soc..

[25] Manbeck G.F., Fujita E. (2015). A Review of Iron and Cobalt Porphyrins, Phthalocyanines and Related Complexes for Electrochemical and Photochemical Reduction of Carbon Dioxide. J. Porphyr. Phthalocyanines.

[26] Zhang X., Wu Z., Zhang X., Li L., Li Y., Xu H., Li X., Yu X., Zhang Z., Liang Y. (2017). Highly Selective and Active CO_2_ Reduction Electrocatalysts Based on Cobalt Phthalocyanine/Carbon Nanotube Hybrid Structures. Nat. Commun..

[27] Birdja Y.Y., Vos R.E., Wezendonk T.A., Jiang L., Kapteijn F., Koper M.T.M. (2018). Effects of Substrate and Polymer Encapsulation on CO_2_ Electroreduction by Immobilized Indium(III) Protoporphyrin. ACS Catal..

[28] Rao H., Lim C.-H., Bonin J., Miyake G.M., Robert M. (2018). Visible-Light-Driven Conversion of CO_2_ to CH_4_ with an Organic Sensitizer and an Iron Porphyrin Catalyst. J. Am. Chem. Soc..

[29] Call A., Cibian M., Yamamoto K., Nakazono T., Yamauchi K., Sakai K. (2019). Highly Efficient and Selective Photocatalytic CO_2_ Reduction to CO in Water by a Cobalt Porphyrin Molecular Catalyst. ACS Catal..

[30] Fukuzumi S., Lee Y.-M., Nam W. (2020). Photocatalytic Redox Reactions with Metalloporphyrins. J. Porphyr. Phthalocyanines.

[31] Wecker M.S.A., Ghirardi M.L. (2014). High-throughput Biosensor Discriminates between Different Algal H_2_-photoproducing Strains. Biotech. Bioeng..

[32] Wecker M.S.A., Meuser J.E., Posewitz M.C., Ghirardi M.L. (2011). Design of a New Biosensor for Algal H2 Production Based on the H2-Sensing System of Rhodobacter Capsulatus. Int. J. Hydrogen Energy.

[33] Lopato E.M., Eikey E.A., Simon Z.C., Back S., Tran K., Lewis J., Kowalewski J.F., Yazdi S., Kitchin J.R., Ulissi Z.W. (2020). Parallelized Screening of Characterized and DFT-Modeled Bimetallic Colloidal Cocatalysts for Photocatalytic Hydrogen Evolution. ACS Catal..

[34] Motz R.N., Lopato E.M., Connell T.U., Bernhard S. (2021). High-Throughput Screening of Earth-Abundant Water Reduction Catalysts toward Photocatalytic Hydrogen Evolution. Inorg. Chem..

[35] Natale P., Brüser T., Driessen A.J.M. (2008). Sec- and Tat-Mediated Protein Secretion across the Bacterial Cytoplasmic Membrane—Distinct Translocases and Mechanisms. Biochim. et Biophys. Acta (BBA)-Biomembr..

[36] Varnado C.L., Goodwin D.C. (2004). System for the Expression of Recombinant Hemoproteins in Escherichia Coli. Protein Expr. Purif..

[37] Reynolds E.W., Schwochert T.D., McHenry M.W., Watters J.W., Brustad E.M. (2017). Orthogonal Expression of an Artificial Metalloenzyme for Abiotic Catalysis. ChemBioChem.

[38] Lelyveld V.S., Brustad E., Arnold F.H., Jasanoff A. (2011). Metal-Substituted Protein MRI Contrast Agents Engineered for Enhanced Relaxivity and Ligand Sensitivity. J. Am. Chem. Soc..

[39] Freudl R. (2018). Signal Peptides for Recombinant Protein Secretion in Bacterial Expression Systems. Microb. Cell Factories.

[40] Lycklama a Nijeholt J.A., Driessen A.J.M. (2012). The Bacterial Sec-Translocase: Structure and Mechanism. Philos. Trans. R. Soc. B Biol. Sci..

[41] Springs S.L., Bass S.E., Bowman G., Noelman I., Schutt C.E., McLendon G.L. (2002). A Multigeneration Analysis of Cytochrome b(562) Redox Variants: Evolutionary Strategies for Modulating Redox Potential Revealed Using a Library Approach. Biochemistry.

[42] Halloran N.R., Banerjee A., Ghirlanda G. (2025). Self-Assembling Peptide–Co-PPIX Complex Catalyzes Photocatalytic Hydrogen Evolution and Forms Hydrogels. Molecules.

[43] Perkins L.J., Weaver B.R., Buller A.R., Burstyn J.N. (2021). De Novo Biosynthesis of a Nonnatural Cobalt Porphyrin Cofactor in *E. coli* and Incorporation into Hemoproteins. Proc. Natl. Acad. Sci. USA.

[44] Weaver B.R., Perkins L.J., Fernandez Candelaria F.O., Burstyn J.N., Buller A.R. (2023). Molecular Determinants of Efficient Cobalt-Substituted Hemoprotein Production in E. Coli. ACS Synth. Biol..

[45] Mazumdar S., Springs S.L., McLendon G.L. (2003). Effect of Redox Potential of the Heme on the Peroxidase Activity of Cytochrome *B*_562_. Biophys. Chem..

[46] Nilsson M., Rydén-Aulin M. (2003). Glutamine Is Incorporated at the Nonsense Codons UAG and UAA in a Suppressor-Free *Escherichia Coli* Strain. Biochim. Biophys. Acta (BBA)-Gene Struct. Expr..

[47] Kramer E.B., Farabaugh P.J. (2007). The Frequency of Translational Misreading Errors in *E. coli* Is Largely Determined by tRNA Competition. RNA.

[48] Salas-Marco J., Bedwell D.M. (2005). Discrimination Between Defects in Elongation Fidelity and Termination Efficiency Provides Mechanistic Insights into Translational Readthrough. J. Mol. Biol..

[49] Ducret A., Quardokus E.M., Brun Y.V. (2016). MicrobeJ, a Tool for High Throughput Bacterial Cell Detection and Quantitative Analysis. Nat. Microbiol..

[50] Schindelin J., Arganda-Carreras I., Frise E., Kaynig V., Longair M., Pietzsch T., Preibisch S., Rueden C., Saalfeld S., Schmid B. (2012). Fiji: An Open-Source Platform for Biological-Image Analysis. Nat. Methods.

[51] Malherbe G., Humphreys D.P., Davé E. (2019). A Robust Fractionation Method for Protein Subcellular Localization Studies in *Escherichia coli*. BioTechniques.

