# Supplementary material for: High-Throughput Screening of Co-Protoporphyrin IX-Binding Proteins for Enhanced Hydrogen Production
Source: Molecules. 2026 Jan 19;31(2):346. doi: 10.3390/molecules31020346 (PMC12844524; doi:10.3390/molecules31020346)
Supplement: Supplementary file 1 [file molecules-31-00346-s001.zip › molecules-4022211-supplementary.pdf]

High-Throughput Screening of Co-Protoporphyrin IX-binding Proteins for Enhanced Hydrogen Production

Nicholas Ryan Halloran, Mohammad Imtiazur Rahman, Abesh Banerjee, Roman Fabry, and Giovanna Ghirlanda

Supplementary Materials

1. Plasmid Construction and Protein Expression

A. Plasmid 1: The Sec fragment together with His_6 tag and TEV cleavage site, encoded in vector pSHTctmaq, is amplified with primers containing NdeI and BamHI (reverse) RE sites (44 °C annealing); the cytochrome b_{562} gene is amplified with primers containing BamHI (forward) and XhoI RE sites (51 °C annealing) (Table 4). The fragments were digested with BamHI and treated with NEB T4 DNA Quick Ligase to obtain Sec-His-Cytochrome b_{562}. The new construct was inserted into pET26b using NdeI and XhoI sites (plasmid 1).

B. Plasmid 2: The plasmid (T7p14_15His_deGFP) used for ChuA transformation contained a 5' 10X-His tag, which needed to be eliminated because ChuA utilizes a 5' signal sequence for expression. Restriction-free (RF) cloning was performed using ChuA primers (Table 4) to delete the 10x-His region using an Agilent GeneMorph II kit. ChuA incorporation and His-tag removal were verified using Sanger sequencing (plasmid 2).

C. Plasmids 1 and 2 were co-transformed into the *E. coli* BL21 DE3 strain for protein expression using kanamycin and ampicillin antibiotics for selection.

D. A single colony from the double antibiotic selection plates was grown in 5 mL of M9 minimal medium (Tables 5 and 6) overnight. The culture was used to inoculate 1 L of culture in M9 medium. Sec-His-cytochrome b_{562} overexpression was induced using 1mM IPTG and the cells were incubated at 37 °C overnight. The cells were then pelleted at 10000x g for 10 min. The pellet was washed three times with 1 mL of 1X M9 salt to eliminate cell debris and metabolites.

E. A few milligrams (2–3 mg) of CoPPIX were dissolved in 100 mM KOH. The solution was diluted to a final concentration of ~5 μM; the accurate concentration was determined by

UV-Vis spectroscopy under anaerobic conditions, using its Soret peak absorption at 417 nm with an extinction coefficient of ε = 143,540 M^{-1} cm^{-1} (as determined by ICP-OES measurements) [1].

F. Cells were incubated with the CoPPIX solution for 30 min at room temperature to allow incorporation into the periplasm via the ChuA receptor.

G. The periplasmic fraction (PF) was isolated using cold osmotic shock with 1 mM $MgCl_2$ [2]. The presence of Co-cytochrome b_{562} in the PF was confirmed by gel PAGE and UV-Vis spectroscopy, which showed a Soret band at 427 nm that shifted to 405 nm upon treatment with sodium dithionite for 30 min at room temperature, consistent with observations of Co-cytochrome b_{562} [1,3].

Notes.

When Sec-His-cyt b_{562} was expressed in LB media in cells co-transformed with plasmids 1 and 2, the cytoplasmic fraction contained Fe^{II}-Sec-His-cyt b_{562}, and the periplasmic fraction contained Co-His-cyt b_{562}. The Sec translocase mechanism, particularly SecB, may be insufficient for binding and maintaining the unfolded form of the protein during overexpression.

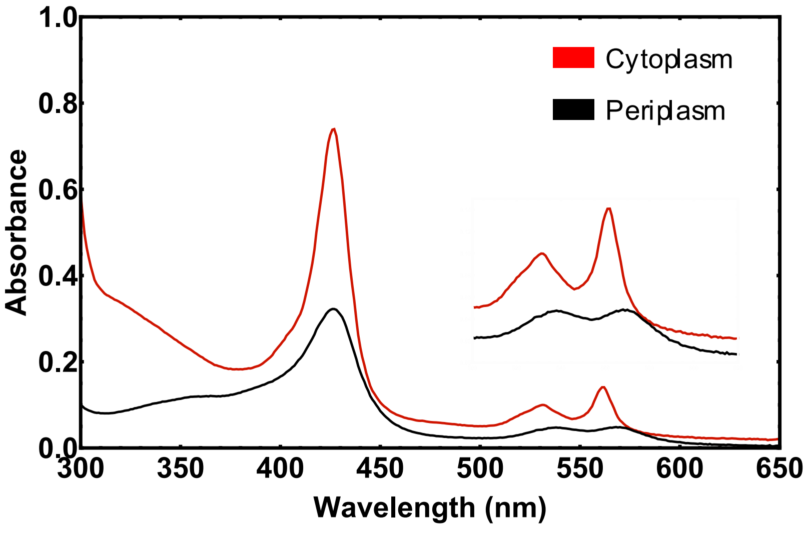

Figure S1. UV-Vis spectra of the cytoplasmic fraction (red) and periplasm extract after incubation with CoPPIX (black).

Table S1: Primer sequences for constructing Sec-His-cytochrome b_{562} and ChuA plasmids.

Primers	Sequence (5'—3')
BamHI Forward	CAGATCCGC**GGATCC**CTGGAAGTACAG
BamHI Reverse	CTGTACTTCCAG**GGATCC**GCGGATCTG
ChuA Forward	GTGATGCGATCCTCTCGTATGTATAT
ChuA Reverse	GATCCTCTCGTATGTATATCTCCTTC
NdeI Forward	CAGGTATTTCATATGAAGATTAAG
XhoI Reverse	ATATATCTCGAGTTAGCGATACTTTTGATGA

Table S2: M9 minimal media composition (1L)

Solutions	Volume (mL)
5x M9 salt stock	200
20% Amino Acid stock	20
20% Glucose stock	10
0.1M $CaCl_2$ stock	1
1M $MgSO_4$ stock	2
dH_2O	767

Table S3: M9 salt stock recipe (5x) for 1 L.

Reagents	Amount (g)
Na_2HPO_4	37.6
KH_2PO_4	15
NaCl	2.5
NH_4Cl	2.5

2. Expression of Co-cytochrome b_{562} (method 2: Biosynthesis of CoPPIX)

The second method for the preparation of Co-Cyt b562 relies on the discovery that native *E. coli* BL21(DE3) can biosynthesize CoPPIX when grown under restricted conditions supplemented with

high concentrations of cobalt and a heme biosynthesis precursor, δ-aminolevulinic acid (δ-ALA). The cofactor is incorporated during the expression of hemoprotein, which sequesters the metal in the form of CoPPIX and limits the toxicity of free cobalt. The ability to biosynthesize CoPPIX results from the promiscuity of ferrochelatases, which catalyze the insertion of metals into free-base porphyrins. Furthermore, BL21(DE3) lacks the RcnA efflux system, and therefore preferentially accumulates cobalt. Recently, the same group optimized the method for expression in rich media supplemented with cobalt. A thorough mutagenesis analysis of the native *E. coli* ferrochelatase EcHemH revealed that the promiscuity of the wild-type enzyme is sufficient for in vivo activity, further highlighting the role of the impaired efflux system [4].

The protocol described below describes the expression of Sec-His-Co-cytochrome b_{562} in M9 minimal medium supplemented with cobalt salts ($CoCl_2$) and a heme biosynthesis precursor (δ-aminolevulinic acid, δ-ALA) [5]. Plasmid 1 was used directly without co-transformation with plasmid 2. Ampicillin was not added to the media.

3.1.1. Expression and Purification

A. Plasmid 1 containing Sec-His-Co-cytochrome b_{562} was prepared as described in Section 3.1.1.A and transformed into *E. coli* BL21 DE3 cells. A single colony was picked from the agar plate and grown in 5 mL of M9 minimal medium containing kanamycin overnight for 16 h.

B. The secondary culture was grown in 5 mL of M9 medium (small culture) by transferring 50 µL of the saturated starter culture. At OD_{600} of 0.2-0.3, M9 media is supplemented with 0.5mM $CoCl_2$ and 0.25mM δ-ALA. Sec-His-cytochrome expression was induced by adding 1mM IPTG. The control culture tubes with/without 0.5mM $CoCl_2$ and 0.25mM δ-ALA were kept for the comparison of the expression. One-liter secondary culture was grown by adding 5mL saturated starter culture in a 2 L Erlenmeyer flask. Sec-His-cytochrome b_{562} was expressed under conditions similar to those in small cultures.

C. The cells were pelleted at 10000x g for 10 min at 4 °C. The pellet was washed with 1 mL (5 ml culture) or 30 mL (for 1 L) of 1X M9 salt three times to eliminate cell debris and metabolites. Cells equivalent to OD_{600} ~2.0 are reserved for whole-cell catalytic activity. The periplasmic fraction (PF) was isolated by cold osmotic shock with 1mM $MgCl_2$. The presence of Co-cytochrome b_{562} in PF was confirmed by SDS-PAGE (single band at the corresponding molecular weight) and UV-Vis spectroscopy (Soret band). The spectrum of PF shows a Soret peak for Co (III) λ_{max} at 430 nm and a distinctive α/β peak at 540/565 nm (Figure 4 A). The Soret peak was shifted to 405 nm after treatment with sodium dithionite for 30 min at room temperature (data not shown).

D. PF from the small culture was saved for catalytic activity measurements. PF from 1 L of culture was further purified by Ni-NTA affinity chromatography using an AKTA pure™ system. Briefly, a 5mL HisTrap column was equilibrated using 5CV (Column Volume) binding buffer containing 20mM sodium phosphate, 500mM NaCl, 20mM imidazole at pH 7.5. The PF samples were stored in the same buffer. The PF sample was loaded into the column and the column was washed with 10CV binding buffer. Proteins were eluted using elution buffer containing 20mM sodium phosphate, 500mM NaCl, 500mM imidazole at pH 8.0. The eluted protein was loaded onto a PD-10 desalting column in 2.5mL fractions and eluted with 3.5mL of 20mM sodium phosphate buffer (pH 7.5). Fifty milliliters of buffer were passed through the column between the protein fractions to wash off any residual salts and re-equilibrate for the next run. TEV protease (protein to TEV; 50:1) was added to the protein with 1mM dithiothreitol (DTT) and 0.5mM ethylenediaminetetraacetic acid (EDTA) overnight at 4°C for complete cleavage. TEV-cleaved Co-cytochrome b_{562} was further purified using Size Exclusion Chromatography (SEC). Proteins were characterized by size on SDS-PAGE and mass-to-charge ratio using MALDI-TOF.

3. Library Design and Expression.

3.1. Cloning.

A. pET26b (+) containing Sec-His-cytochrome b_{562} was used for library generation (Figure 2).

Table S4. Primer sequences for error-prone PCR.

Primers	Sequence (5'—3')
Forward	AGCGGCGAGAACCTGTACTTCCAG
Reverse	TGGTGGTGCTCGAGTCAGCGATA

B. The purified products of RF cloning reaction were digested with *dpnI* to eliminate residual parental DNA, and 1.5 μL of DNA was transformed into NEB 5-alpha *E. coli* cells (NEB, C2987H) following manufacturer protocol (https://www.neb.com/en-us/protocols/0001/01/01/high-efficiency-transformation-protocol-c2987). The cells grown in 1mL outgrowth SOC medium were plated on agar plates containing kanamycin and placed in an incubator oven overnight. Each plate contained 1000+ colonies and 50 ng of the library DNA. A transformation frequency of roughly 1.2* 10^5 was calculated (https://www.sciencegateway.org/tools/transform.htm). Ten colonies were randomly selected and sequenced (Genewiz) to estimate the frequency of mutations. A mutation frequency of 2-4 was observed based on sequencing, and all 10 colonies contained at least two mutations.

C. Colonies from all plates were combined into a 15mL falcon tube by washing off with fresh LB medium. The plasmid containing the Sec-His-cytochrome b_{562} library was purified using the Qiagen Spin Miniprep Kit. DNA yield and purity were verified using a NanoDrop 2000 spectrophotometer. Library DNA was stored at -80°C for future use. (Plasmid L)

D. Library DNA (Plasmid L) is co-transformed with ChuA plasmid (Plasmid 2) into *E. coli* BL21 DE3 competent cells ((https://www.neb.com/en-us/protocols/0001/01/01/transformation-protocol-for-bl21-de3-competent-cells-c2527) and the cells plated on agar containing kanamycin and ampicillin. The plate was stored at 4 °C for one week prior to screening. Re-transformation was performed weekly to obtain viable cells.

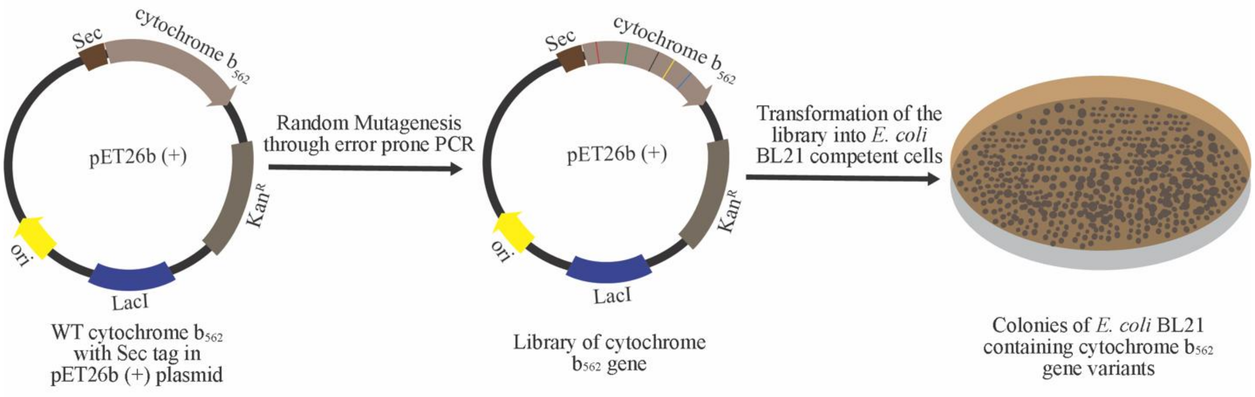

Scheme S1. Library preparation.

3.2. Library Expression (method 1)

A. Individual colonies containing plasmids L and 2 were picked from the agar plate and grown in 5 mL of M9 minimal medium containing kanamycin and ampicillin overnight for 16 h. Colonies were grown in a deep-well 96-well plate in 500 µL medium.

B. Sec-His-cytochrome b_{562} overexpression was induced using 1 mM IPTG and the cells were incubated at 37 °C overnight. 1mL was removed from each culture and centrifuged at 10,000 g for 5 min.

C. The supernatant was removed and the pellet was washed twice with 1mL of M9 salt before spinning down at 10,00x g for 3 min.

D. The cells were resuspended in M9 medium containing 5 µM Co-PPIX for 30 min at room temperature to allow uptake of CoPPIX into the periplasm via the ChuA transporter. PF was isolated using cold osmotic shock in either an Eppendorf tube or deep-well plate. The porphyrin content bound to cytochrome b_{562} was monitored using cuvettes in a spectrometer or plate reader. The basal concentration of porphyrin was determined by monitoring the Soret band at 427 nm. The protein concentration was measured independently at 280 nm.

3.2. Library Expression (method 2)

A. Individual colonies containing Plasmid L were picked from the agar plate and grown in 5 mL of M9 minimal medium containing kanamycin overnight for 16 h. Colonies were grown in a deep-well 96-well plate in 500 µL medium.
B. Sec-His-cytochrome b_{562} overexpression was induced using 1 mM IPTG and the cells were incubated at 37 °C overnight. 1mL was removed from each culture and centrifuged at 10,000 g for 5 min.
C. The supernatant was removed and the pellet was washed twice with 1mL of M9 salt before spinning down at 10,00x g for 3 min.
D. The cells were resuspended in M9 medium. PF was isolated by cold osmotic shock (5.2.1.C), either in an Eppendorf tube or in a deep-well plate. The concentration of Co-PPIX-bound cytochrome b_{562} was determined by measuring the absorbance of the Soret band at 427 nm. The protein concentration was measured independently at 280 nm.

Notes

A. The mutation frequency can be regulated by controlling the number of cycles in error-prone PCR and the initial amount of template DNA. The purity of template DNA was assessed by measuring the DNA content at 260 nm and calculating the 260/280 ratio, which was close to 1.8.
B. High-throughput screening should be performed on the day of the library expression.

4. Preparation of WO_3/Pd plate

1. Wash a glass plate thoroughly with 20% ethanol and water

2. Dissolve the WO_3 nanoparticles in 2 M HCl to a concentration of 1 mg/mL. Tape the glass plate onto the lab bench using electric tape. Disperse the WO_3 suspension across the top of the plate. To ensure uniform distribution of the WO_3 suspension, use a Pasteur pipette to slide across the length of the plate.

3. Allow the plate to dry at room temperature for 15 minutes before placing it in a oven at 300°C for 30 minutes. After 30 minutes, allow it to cool back to room temperature.

4. Prepare a Pd-TFA solution by dissolving it in 2 (M) HCl at a concentration of 100 μg/μL. Add 15 μL of this solution to the center of the WO_3 plate. Cover the plate with a plastic lid to ensure uniform distribution of the Pd-TFA solution across the WO_3 plate via capillary action and to prevent premature drying of the solution by airflow.

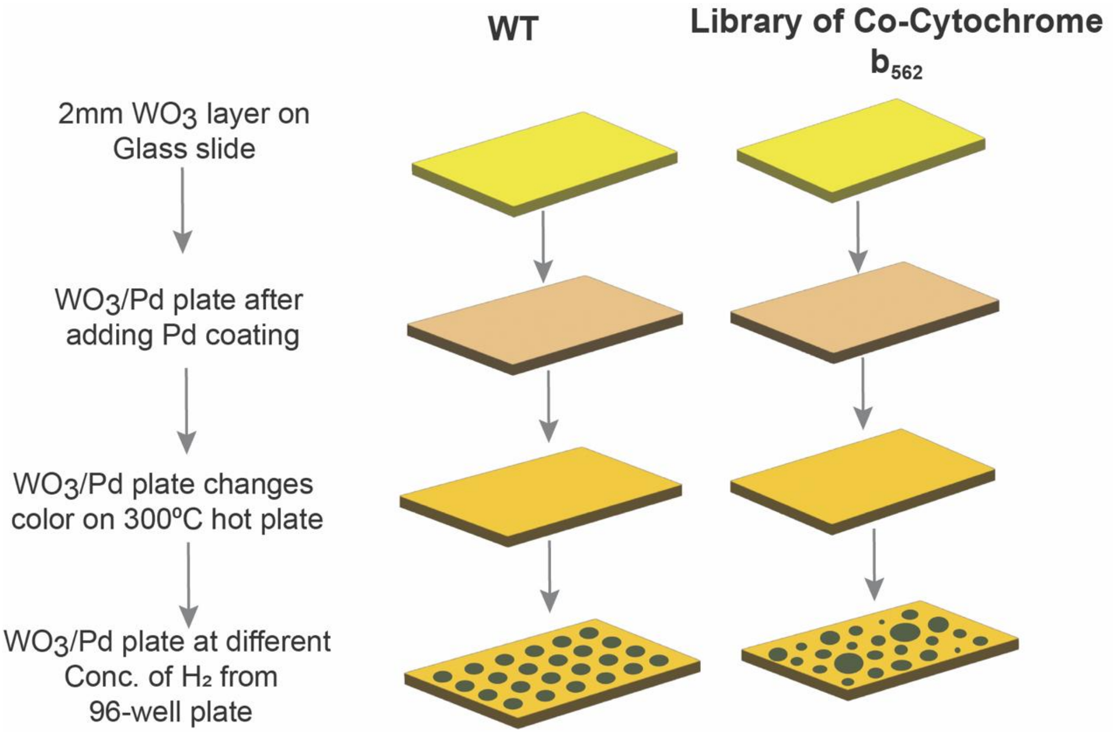

Scheme S2. Plate assembly.

4.1. Standardization of plates.

Compared to the wild-type, the mutant 25 variant showed higher H_2 activity from whole cells, periplasm extract, and HPLC-purified protein, as seen by the size of the dark spot on the WO_3/Pd plates. The differences in background color were due to the thermochromic nature of WO_3, and the pictures were taken at slightly different times after heating at 300 °C. The yellow color, as seen in the pure protein background, occurs when WO_3 is heated, and fades quickly to beige when sitting at room temperature. Although the color of the background changed, the size of the H_2 induced spot remains constant during cooling.

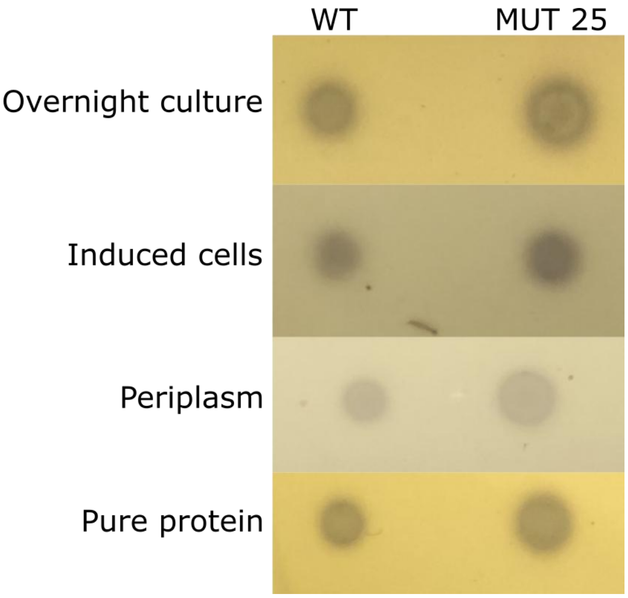

Figure S2. Wo3/Pd plates comparing H_2 production by wild-type and mutant 25 cytochrome b_{562}.

5. Mutational analysis

DNA from Mutant 25 was isolated and sequenced to identify mutations associated with enhanced activity. The analysis revealed a total of seven point mutations within the DNA sequence, four of which were silent. A serine residue was inserted at position 19 in the Mut 25 sequence for downstream applications, a feature absent in the wild type (WT). Sequence alignment indicated two amino acid modifications at positions A39V and D40N.

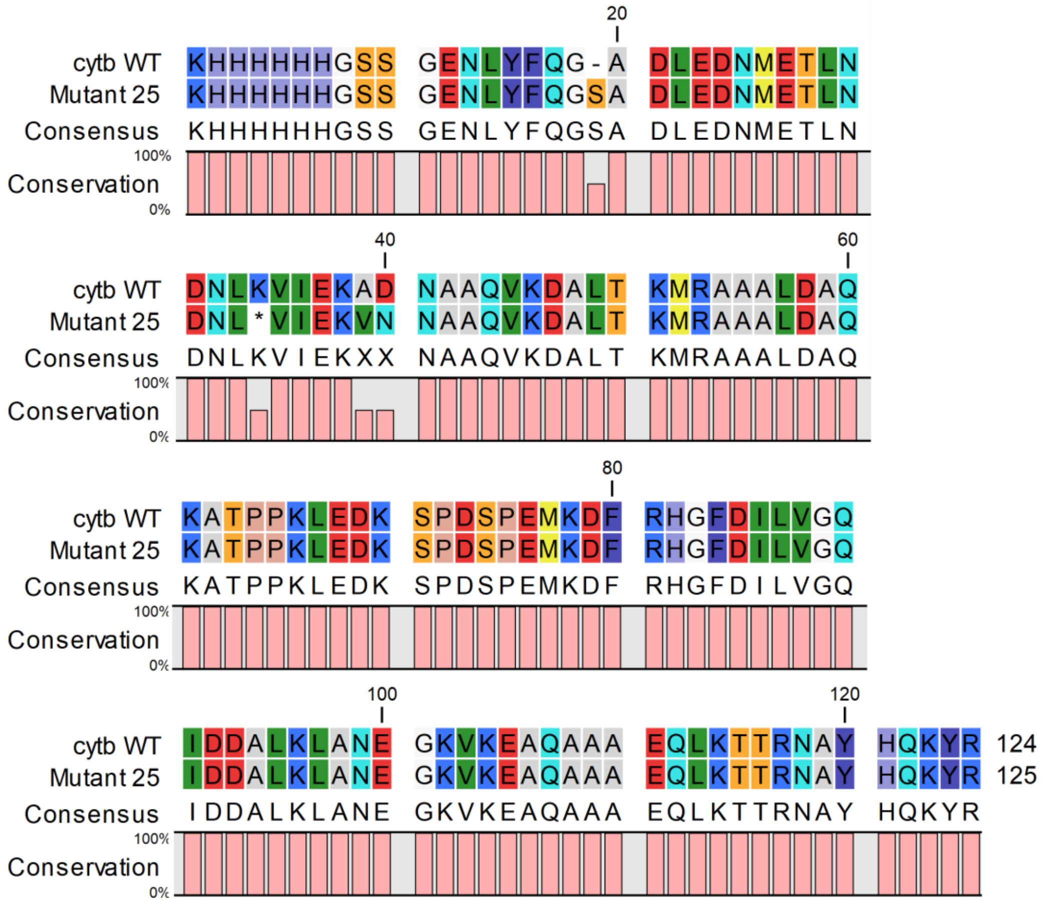

Figure S3. Amino acid alignment of wild-type cytochrome b_{562} and Mut25 variants.

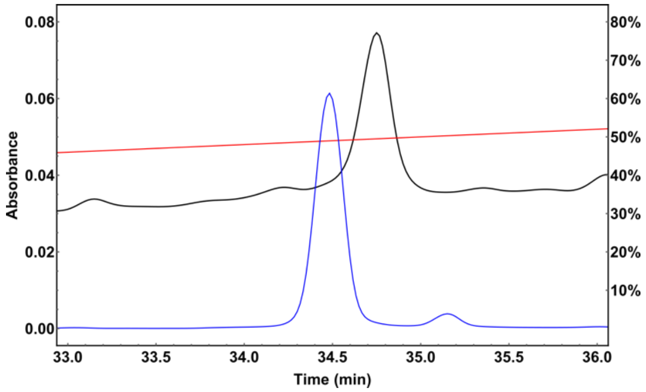

Figure S4. HPLC elution profile of WT (blue) and Mut 25 (black); percent solvent B composition is shown as a red line (Y axis, right).

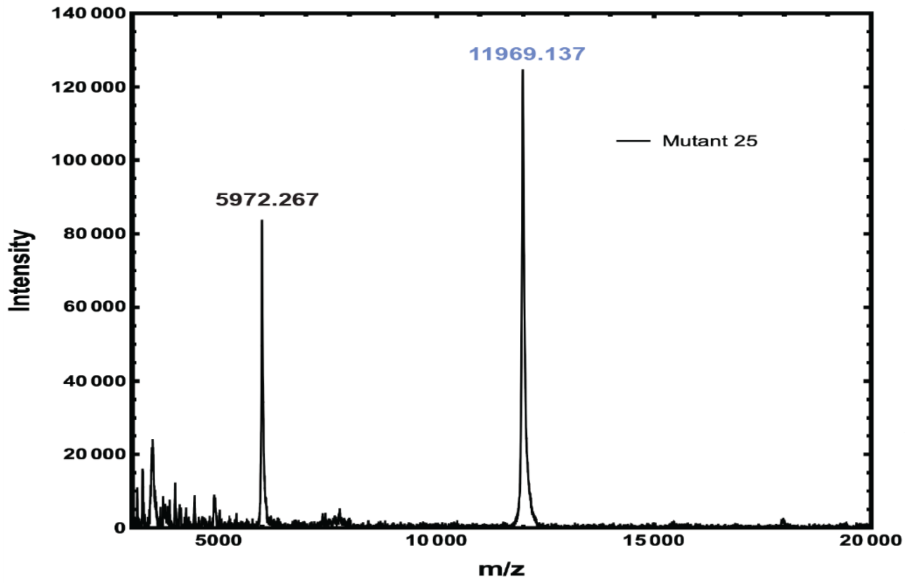

Figure S5. MALDI analysis of HPLC purified Mut25.

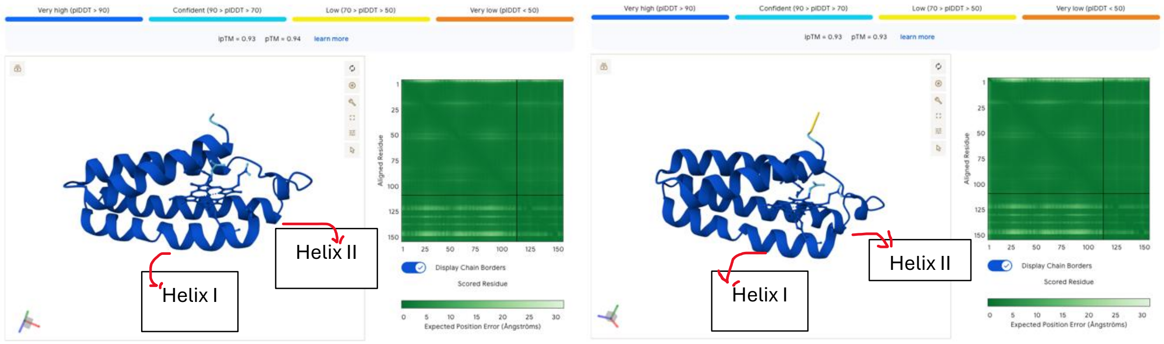

Figure S6. AlphaFold3 models of WT cytb_{562} (left) and Mut25; both structures are predicted with high confidence (pIDDT >90).

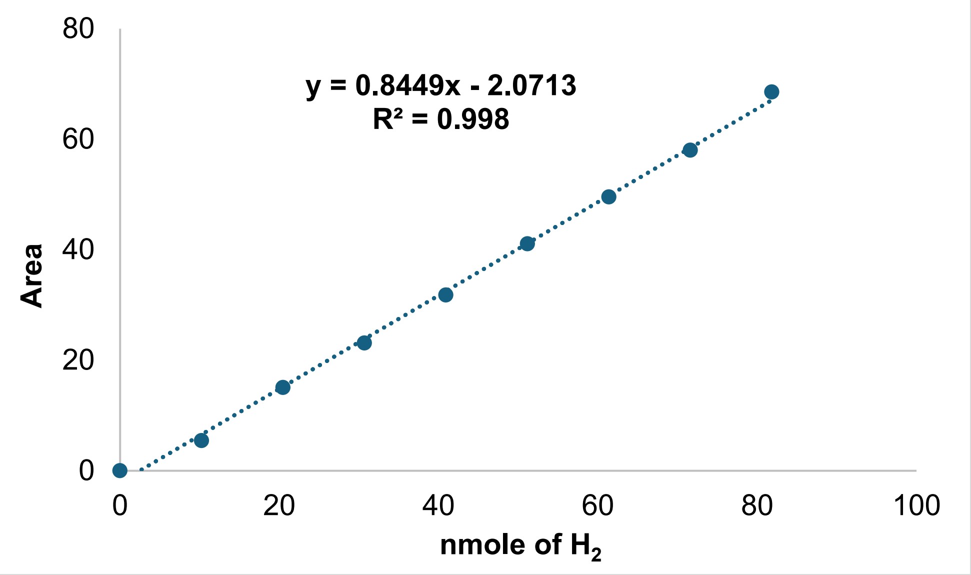

Figure S7. Standard curve for GC analysis of H_2 production, generated by injecting increasing amounts of hydrogen. R^2 = 0.998.

References

1. Alcala-Torano, R.; Halloran, N.; Gwerder, N.; Sommer, D.J.; Ghirlanda, G. Light-Driven CO2 Reduction by Co-Cytochrome B562. *Frontiers in Molecular Biosciences* **2021**, *8*.
2. Malherbe, G.; Humphreys, D.P.; Davé, E. A Robust Fractionation Method for Protein Subcellular Localization Studies in Escherichia Coli. *BioTechniques* **2019**, *66*, 171–178, doi:10.2144/btn-2018-0135.
3. Sommer, D.J.; Vaughn, M.D.; Clark, B.C.; Tomlin, J.; Roy, A.; Ghirlanda, G. Reengineering Cyt B562 for Hydrogen Production: A Facile Route to Artificial Hydrogenases. *Biochimica et Biophysica Acta (BBA) - Bioenergetics* **2016**, *1857*, 598–603, doi:10.1016/j.bbabio.2015.09.001.
4. Weaver, B.R.; Perkins, L.J.; Fernandez Candelaria, F.O.; Burstyn, J.N.; Buller, A.R. Molecular Determinants of Efficient Cobalt-Substituted Hemoprotein Production in *E. coli*. *ACS Synth. Biol.* **2023**, *12*, 3669–3679, doi:10.1021/acssynbio.3c00481.
5. Perkins, L.J.; Weaver, B.R.; Buller, A.R.; Burstyn, J.N. De Novo Biosynthesis of a Nonnatural Cobalt Porphyrin Cofactor in *E. coli* and Incorporation into Hemoproteins. *Proceedings of the National Academy of Sciences* **2021**, *118*, e2017625118, doi:10.1073/pnas.2017625118.
